# The Finishing Space Value for Shooting Decision-Making in High-Performance Football

**DOI:** 10.3390/sports12080208

**Published:** 2024-07-30

**Authors:** Nelson Caldeira, Rui J. Lopes, Duarte Araujo, Dinis Fernandes

**Affiliations:** 1 CIPER, Faculdade de Motricidade Humana, Universidade de Lisboa, 1499-002 Cruz Quebrada, Portugal; coach.caldeira@gmail.com; 2 ISCTE-Instituto Universitário de Lisboa, 1649-026 Lisboa, Portugal; rui.lopes@iscte-iul.pt; 3 Instituto de Telecomunicações, 1049-001 Lisboa, Portugal; 4 Independent Researcher, 3810-193 Aveiro, Portugal

**Keywords:** affordances, degeneracy, performance, team synergies, Voronoi diagrams

## Abstract

Football players’ decision-making behaviours near the scoring target (finishing situations) emerge from the evolving spatiotemporal information directly perceived in the game’s landscape. In finishing situations, the ball carrier’s decision-making about shooting or passing is not an *individual* decision-making process, but a *collective* decision that is guided by players’ perceptions of match affordances. To sustain this idea, we collected spatiotemporal information and built a model to quantify the “Finishing Space Value” (FSV) that results from players’ perceived affordances about two main questions: (a) is the opponent’s target successfully *reachable* from a given pitch location?; and (b) from each given pitch location, the opposition context will allow *enough* space to shoot (low adversaries’ interference)? The FSV was calculated with positional data from high-performance football matches, combining information extracted from Voronoi diagrams (VD) with distances and angles to the goal line. FSV was tested using as a reference the opinion of a “panel of expert” (PE), composed by football coaches, about a questionnaire presenting 50 finishing situations. Results showed a strong association between the subjective perception scale used by the PE to assess how probable a shot made by the ball carrier could result in a goal and FSV calculated for that same situation ($R^{2}=0.6706$). Moreover, we demonstrate the accuracy of the FSV quantification model in predicting coaches’ opinions about what should be the “best option” to finish the play. Overall, results indicated that the FSV is a promising model to capture the affordances of the shooting circumstances for the ball carrier’s decision-making in high-performance football. FSV might be useful for more precise match analysis and informing coaches in the design of representative practice tasks.

## 1. Introduction

Being a low-score game, a goal in football is a “critical event” [1] with enormous influence on the match event’s succession [2] and in its final result [3]. In the present paper, we studied the decision-making processes involved in finishing situations, which are of paramount importance for matches’ final result.

As in other invasion games [4,5], both teams in a football match have mutually exclusive goals [4,6]. This feature shapes a collective context where players of a given team need to cooperate to achieve common purposes [7,8]. When attacking, one of the main objectives of a team is to search for a clear opportunity to shoot at the opponent’s goal [6,9]. In that game’s moment, the decisions and actions involving the player that “controls” the ball (the “ball carrier”) are particularly important. However, from an ecological dynamics perspective, psychological processes such as perception, action and decision-making should be understood at the environment–athlete system level [10], where perception and action are tightly intertwined and cannot be separated [11]. Accordingly, the behaviour of the “ball carrier” is contingent with the dynamics of the interaction between the two teams—as every player’s action is constrained by the actions of all other players (teammates and opponents)—that shape the game’s landscape [12]. In this view, the game landscape reveals affordances (i.e., “opportunities for action”) [11,13] that guide players’ decisions [14], informing what (and how) actions are possible for each player [15,16].

From this ecological dynamics perspective, players’ actions are thus *joint* or coordinated actions [13] leading to the appearance and disappearance of match affordances that are collectively perceived (e.g., opening for a pass) [17,18]. Consequently, the action of every individual player cannot be seen as isolated or pre-determined (e.g., by the coach before the match, attributing a particular static role to a given player). It should be seen, instead, as the result of ongoing team synergies [19,20] that are continuously formed and dissipated as the game landscape of affordances changes [21]. Establishing a causal circularity typical of synergies [22], the behaviours of each player influence and are influenced by the behaviours of the others. In fact, even if perceiving an affordance in the performance environment is predicated on each player’s action capabilities [21,23], the ball carrier’s decision-making on whether to shoot or not (e.g., passing to a better-positioned teammate) emerges from their perception of the game’s landscape that is shaped by the (joint) action of all elements on the pitch [24].

Therefore, in this paper, we posit that players decisions in finishing situations emerge from players’ perception of game’s landscapes and its possibilities for action, i.e., on the game’s *shared* affordances that could invite players to act (e.g., to shoot or to pass) [11,17,18]. Moreover, we argue that the information captured from the match positional data can be used to quantify players’ *affordances* in football finishing situations [25]. Additionally, more than simply considering action possibilities as binary categories (e.g., to shoot or not at the opponent’s goal), we agree with Franchak and Adolph [26] when they propose the quantification of affordances through *probabilistic functions* (or *affordances functions* [26]) to describe the *likelihood of success* for every parameter of a given environment [26].

In fact, when football players perceive shooting possibilities, they consider how *reachable* it is from their *location* on the pitch [13,27] as an affordance that could be assessed through the computation of the *success ratio* of a shot made from a given pitch location. This is performed, for example, by Pollard and colleagues [28] and Link and colleagues [29] that use two parameters (distance and angle) to estimate the probability of a shot, resulting in a goal. Despite the differences between these models, they converge in the net result that the closer the ball carrier is to the opponent’s goal, the greater the probability of scoring (see the heatmaps in Appendix A, Figure A4 and Figure A5). An identical concern with the probability to score is also found in the main purpose of several models that are usually known as *expected goal* (xG) models [30,31,32,33,34,35,36], which typically express the *likelihood of success* of a shot made from a given pitch location.

However, in many finishing situations, an affordance to shoot from a given pitch location can cease to exist due to the actions of the opposition team. This is particularly evident when the opponents closest to the “ball carrier” try to prevent her or him from having *enough* space to shoot. This is considered differently in several models; for example, Pollard and colleagues (p. 54, [28]) address the question of “whether or not the person taking the shot had space” with a binary system that is “quantified by 0 if there was an opponent within one metre, and 1 if not”. That is, as long as there was an opponent less than a meter away, this model completely excludes the chances of scoring a goal. Similarly, the shot “dangerousity” model proposed by Link and colleagues [29] includes a “Pressure” parameter to consider how the distance to the nearest opponents can disturb a possible shot. This concern with the opposition context that conditions the emergence of a shooting affordance is precisely one shortcoming of several xG models. Albeit under the same name (xG), there are significant computational differences between models. To our knowledge, there is only one xG model, proposed by Rowlinson [34], that, using positional data, integrates some information about the opposition context. This model involves the computation of Voronoi diagrams (VDs) to assess each *Voronoi cell* (VC) as a measure of the *free space* around some players. However, this model only integrates the Voronoi area (VA) of the the ball carrier and the opposing goalkeeper.

To overcome the lack of information on the game’s landscape that is common to xG models, we propose a “Finishing Space Value” (FSV) model that was somehow inspired by the “Expected Possession Value (EPV)” model proposed by Fernandez and colleagues [37,38] and previous basketball studies of Cervone and colleagues [39]. The EPV “incorporates the dynamics of the 22 players and the ball through tracking data” (p. 1389, [38]) to estimate the likelihood of a team scoring or conceding the next goal at any time instance. However, in the EPV, there is a completely different approach to compute players’ *free spaces* around players. While in the proposed FSV we use VDs to assess the “free space” of each player, the computation of Fernandez and colleagues [38] is based on players’ ”reachability surface”, i.e., the pitch surface that a player can cover in a certain *time lag* (in this case, in one second), given their direction and velocity vector. We posit that “free spaces” resulting from the distances among players over time can be more accurately captured through VDs and their derived metrics than from models using “reachability surface” [38,40] with a fixed time lag. This difference is especially important in “finishing situations” where players increase the simulation of trajectories [41,42], thereby implying a constant update of their predicted trajectories [43].

In this context, we propose a new model to quantitatively assess, from positional data captured in high-level football game’s landscapes, the *affordances* that possibly guide players’ actions in finishing situations. The FSV model is designed for very specific shooting situations: in open-plays (e.g., set-pieces are not included) and in shots made with feet (e.g., shots made with the head are not included). Being grounded in the ecological dynamics theoretical framework [10,11], the FSV is composed of two main *affordance functions* [26] aiming to quantify (a) how *successfully reachable* can be the opponent’s goal when a shoot it is made from a given pitch location (considering the distance and angle to the opponent’s goal; see Section 2.1.1) and (b) how *broad* it is the space to shoot from that location [13,17] (assessed using VD of all players on the pitch; see Section 2.1.2).

Thus, the main purpose of this study is to propose and test if the FSV model is able to combine in a single value the two mentioned *affordance functions*, capturing the *value* of occupying a certain location and space on the pitch in football finishing situations. To this end, we compare the results of the FSV model with the subjective opinion of expert football coaches about what they consider to be the “best option” in a set of game situations.

## 2. Materials and Methods

### 2.1. Data Sources

Two data sources are used in this paper with different aims: (a) a positional and notational database used to estimate different parameters of the FSV model (pitch location and free space valuation, as described in Section 2.1.1) and (b) a database with the subjective opinion of a “Panel of Expert” (PE) football coaches about 50 finishing situations (see Section 2.1.2).

#### 2.1.1. Positional and Notational Data Source for Computing FSV Model Parameters

The FSV model parameters were computed from positional and notational data obtained from 283 games from the Ligue 1 and Champions League competitions, played between 2016 and 2020. Positional data refer to the longitudinal and lateral coordinates of the ball and all players on the pitch. Notational data refer to events during the match (e.g., passes, shots), their outcome, and the players involved. This database was provided by the company STATS and computed using their semi-automatic tracking systems (as validated in [44]). The parameters obtained from these data pertain to two different aspects of the model:

(a) Assign a value to pitch locations. This value corresponds to the probability of scoring from a shot made from that location (defined by distance and angle to goal). A total of 5294 shots made with the players’ feet and from open-play situations were observed, from which 543 goals were scored.
(b) Assign a value to the free space around each player. Computed as the relative value of this space compared to the average value for the same location obtained from a subset of 20 football matches of the database, randomly selected from the 2019–2020 season.

#### 2.1.2. Affordances Assessment by Football Coaches

The affordance assessment by the FSV model was tested against the opinion of a PE composed of 10 Portuguese professional football coaches, all former players at different levels and holding a UEFA PRO license with a minimum of 10 years of high-level football coaching experience in the Portuguese first league.

The PE opinion was obtained through a questionnaire run between August and September of 2022. The questionnaire was applied to 50 finishing situations randomly selected from the set of high-performance football matches from the 2019–2020 season described in Section 2.1.1.

The 50 situations are selected from the 311 shots made in these 20 matches with the players’ feet, in open-play situations, and where the ball carrier had, at least, two other teammates as passing possibilities in the offensive last third of the pitch.

**Figure 1:**
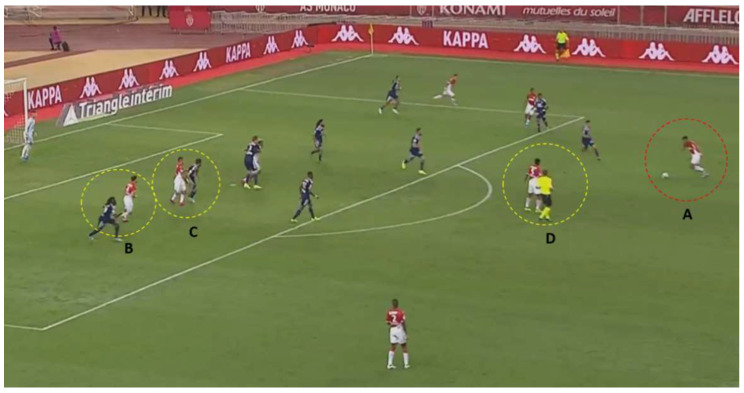
Illustrative image of a finishing situation presented in the survey to the “panel of expert” football coaches. Player “A” is the ball carrier who shoots and the players marked B, C and D are his colleagues that we consider as possible passing options.

In the survey, each situation was illustrated by an image (as exemplified by Figure 1) corresponding to the instant of the shot, wherein players are identified with letters A (the ball carrier) and B to D (the ball carrier’s teammates that are closer to the opponent’s goal). Alternative options (such as dribbling the ball or passing to other players) are represented by option E.

For each of the 50 situations, two questions were posed independently to each PE element:

In this image, player A took the decision to shoot at the goal.

(a) Please evaluate, on a scale from 1 to 10 (where 1 is “not at all likely” and 10 is “highly likely”), what you consider to be the *probability* to score a goal from that specific shot of player “A”.
(b) Do you think that player A chose “the best option” in that situation, or would it be preferable to pass to one of his teammates (B, C or D)? If you consider that the play should not be finished immediately, as neither player A nor any of his colleagues (B, C or D) are in a good position to score immediately (i.e., by shooting or through a single-assistance pass), please choose option “E”.

### 2.2. Data Processing

The FSV quantification for each player was calculated for each finishing situation (see example in Figure 1). For each player (A, B, C and D), the FSV model integrates three parameters: (a) the “Player Location” (PL); (b) the player “Relative Voronoi Area” (RVA); and (c) the player “Relative Voronoi Position” (RVP). The PL is computed from the location of each player, considering the distance and angle of each player to the centre of the opponent’s goal line. The two other parameters, RVA and the RVP of each player, are obtained from the VDs computed from the distances among all players on the pitch. The FSV is defined in arbitrary units (AUs), and calculated by the multiplication of these three parameters, i.e.,

FSV=PL·(RVA·RVP)

**Figure A3:**
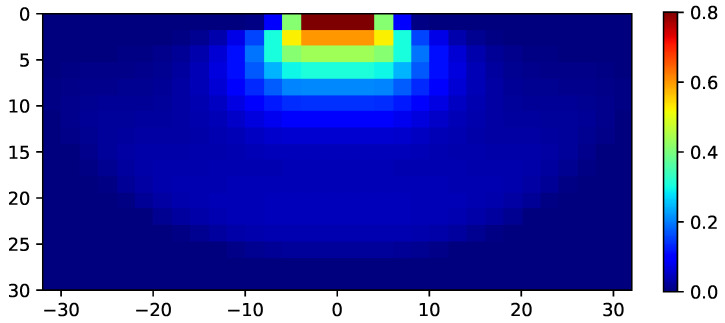
Heatmap with the probability of scoring calculated by the PL component of our FSV model (see Section 2.2.1).

Therefore, the parameters used in the FSV computation correspond to the following:

1. The PL, which is computed as the probability of achieving a goal from a shot at a given position (see Figure A3 in Appendix A), considering two sub-components: (a) the distance and (b) the angle of each player in relation to the opponent’s goal (described in Section 2.2.1).
2. The RVA, capturing the space around each player in a VD, corresponding to the respective cell area and considering its specific location in the effective playing space (EPS) (described in Section 2.2.2).
3. The RVP, depending on the player’s distance to their nearest opponent towards the goal line (described in Section 2.2.3).

For all three parameters, a similar process of fitting a polynomial curve to the database data is used, as illustrated in Figure A1,Figure A2,Figure A3,Figure A4,Figure A5,Figure A6,Figure A7,Figure A8,Figure A9 and Figure A10 in Appendix A.

#### 2.2.1. The Player Location (PL)

**Figure 2:**
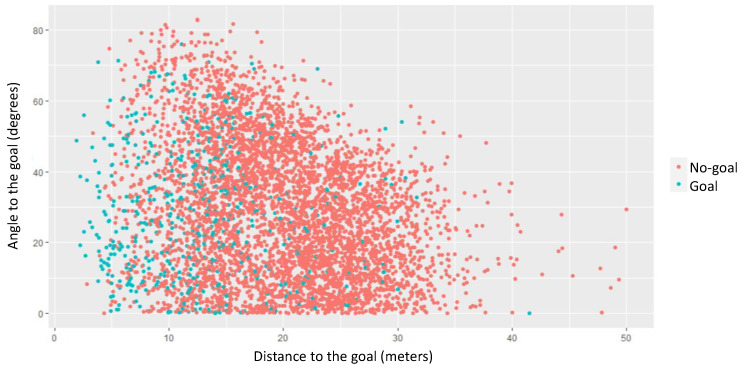
Shots with (blue) and without (red) scoring according to distance and angle to goal.

The PL quantifies the probability to score in a shot made from a specific location on the pitch, considering the *distance* and the *angle* to the centre of the goal line. Figure 2 shows the outcome of the 5294 shots in the database (543 goals scored in blue (10.26%), 4751 shots without goal in red) given the distance and angle to the goal.

These data were used to estimate the parameters of a polynomial equation for the scoring probability given the positional coordinates (distance and angle) of a player. Thus, the probability (in %) to score varies according to the value *x* corresponding to the PL distance (PLd), in meters, to the centre of the opponent’s goal line, as follows:

$$PLd=-0.0107x^{3}+0.7061x^{2}-15.3620x+115.1100$$

Figure 2 also shows how the angle to the centre of the goal influences the probability to score. In this case, the probability (in %) to score (PLa) depends on the PL angle (*x*), in degrees, to the centre of the opponent’s goal line, as defined by

$$PLa=-0.0129x^{2}+0.0347x+79.7020$$

The PL component was then calculated from the multiplication of these two polynomial equations obtained from the players’ distance and angle to the centre of the opponent’s goal line:

PL=PLd·PLa

**Figure A4:**
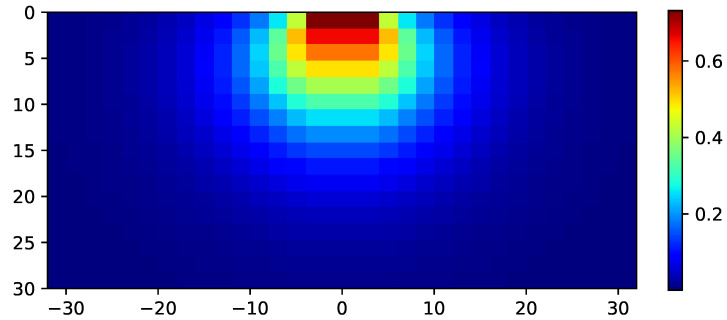
Heatmap with the probability of scoring in a shoot made from a given pitch location, calculated by the model of Pollard and colleagues [28]. They concluded that for each additional yard between the player and the goal, the probability of scoring decreased by 15%, whilst for each angle degree, there was a decrease of 2%.

**Figure A5:**
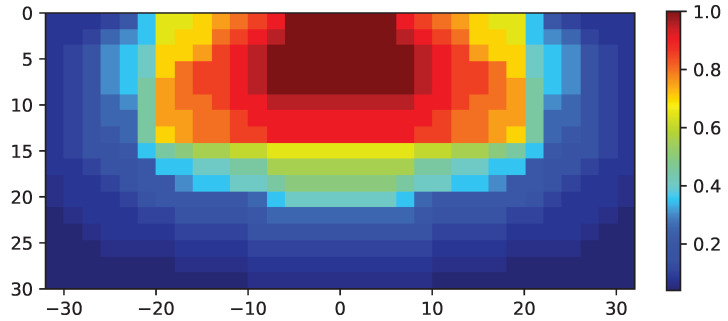
Heatmap with the probability of scoring calculated by the "zone" component of the model of Link and colleagues [29].

The PL, expressed as a probability, can be visualized in Figure A3 of Appendix A and compared with similar approaches made by Pollard and colleagues [28] (Figure A4) and Link and colleagues [29] (Figure A5).

#### 2.2.2. The Player Relative Voronoi Area (RVA)

**Figure 3:**
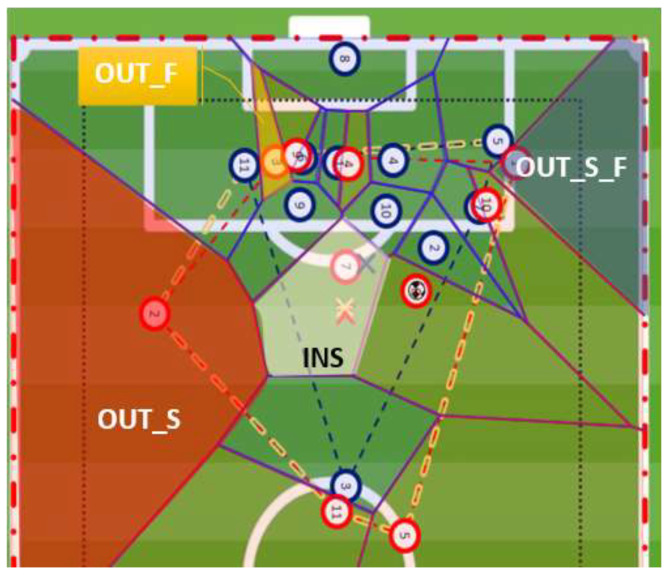
Player’s VC in four different regions of the EPS. Red circles for “in ball possession” team players and blue circles for the“out of ball possession” team players.

To assess players’ context in the game’s landscape, the VA corresponding to each player was calculated from the positional data of the matches’ database, using a set of computer routines in Excel (VBA) to automate the set of procedures described by Kim [45]. The VA of each player is defined by the absolute area of each VC (in m^2^). However, players’ absolute VA must be placed in the proper context. In fact, the circumstance that a player is *inside* or *outside* the EPS [46] strongly influences the VA absolute value. As exemplified in Figure 3, in a VD, it is possible to identify four possible regions of the EPS (yellow dashed line) where a player can be in a given instant:

(a) VC inside the EPS (INS), i.e., that does not make contact with any of the outer lines of the pitch or with the goalkeepers’ cells (e.g., the white shaded cell in Figure 3).
(b) VC outside and in front of the EPS (OUT_F), which makes contact only with the opposing goal line or the opposing goalkeeper’s VC (e.g., the yellow shaded cell in Figure 3).
(c) VC outside the EPS, which makes contact only with the pitch sideline(s) (OUT_S) (e.g., the red shaded cell in Figure 3).
(d) VC outside the EPS that makes contact, simultaneously, with the opposing goal line or the cell of the opposing goalkeeper (front) and at least one of the pitch outside the lines (OUT_S_F) (e.g., the blue shaded cell in Figure 3).

The modulation effect of the RVA parameter is obtained by comparing the *actual* Voronoi area (VA) of each player to the *expected* Voronoi area (*VAe*) for that pitch location. Using the average values of players’ VA in the matches database, it was possible to calculate the players’ *VAe* by considering players’ distance to the centre of the opponent’s goal line (*x*) in four different regions of the EPS (as shown in Figure 3). Table 1 and Table 2 present the equation estimated for the players’ *VAe* for each of the four EPS regions. Different equations are obtained for the *ball carrier* (BC) and for his teammates *without the ball* (NB) as these correspond typically to different relations between the ball carrier and other players (e.g., ball carrier pressure).

As our purpose was to use VDs to measure if players had *enough* space to shoot, their VA absolute values measured in a given instant had to be transformed in relative values. Accordingly, we assessed if players’ VA at each shot, with its distance to the goal and EPS zone, were smaller or larger than the *typical* (expected) VA at that distance and EPS zone. To this end, we *divided* players’ *actual* VA measured in each finishing situation (VA), by the VA that a player in the same contextual circumstances was *expected* to have (VAe). Thus, the RVA is calculated by

$$RVA=\frac{VA}{VAe}$$

#### 2.2.3. The Player Relative Voronoi Position (RVP)

**Figure 4:**
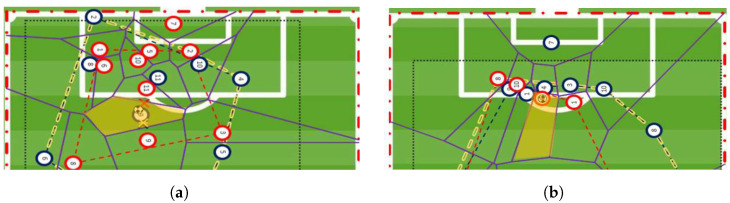
VDs with different distances to the nearest defender. (**a**) Larger distance to closest defender; (**b**) smaller distance to closest defender.

The third parameter of the FSV model was introduced to take into account the player’s position within the VC. For the ball carrier or his/her teammates, the perception that they have *enough* space to shoot is not only a consequence of their VA but also of their relative position inside the respective cell. This is exemplified in the two diagrams in Figure 4, where two very different positions are represented in VC with almost identical areas (in m^2^).

Figure 4 shows that, although in both situations the VA (in m^2^) of the ball carrier (highlighted in yellow) is similar (around 40 m^2^), the distance from the nearest opponent (between him and the opponent’s goal) is different in Figure 4a (larger) and Figure 4b (smaller). This means that the space to shoot for the player in Figure 4a can be bigger than the one for the player in diagram Figure 4b. Thus, even in identical regions of the EPS (inside), the player’s positioning within the VC is influenced by his distance to their nearest opponents. To assess players’ space to shoot, it is important to consider the distance between each player and the nearest opponent towards the goal line. The differences in the players’ RVP are expressed mathematically by

RVP=max(0,ln(d))

where ln(d) is the natural logarithm of the distance, and *d* is the distance between each player of the in-possession team and his nearest opponent. To exemplify the impact of RVP in the FSV model, when a player has an opponent at a distance smaller than 1 m, the application of the natural logarithm will have a negative value, leading to a FSV of 0. This reflects the high pressure potentially placed by that opponent in that specific situation, thus reducing the value of the space to finish the play with a shot.

### 2.3. Statistical Analysis Methods

The way in which the FSV model captures the affordances to shoot in finishing situations was assessed by comparing it with the opinions of a “Panel of Expert” (PE) football coaches. In other words, the probability of coaches choosing each option in each finishing situation was compared with the respective FSV quantification for each player in each of the presented finishing situations (see example in Figure 1). Three statistical tools were used:

(a) A linear regression between the FSV and the scale used by the PE, on how probable a shot made from player A (i.e., the ball carrier) in each situation could result in a goal.
(b) The Gwet AC1 statistic [47,48], to assess the inter-rater reliability coefficient, i.e., the degree of agreement among the coaches of the PE when they choose one option of $C_{M}=\left\{A,B,C,D,E\right\}$ for each of the 50 finishing situations of the questionnaire. The Gwet AC1 statistic is computed using
  (1) $$AC1=\frac{p_{a}-p_{e_{\gamma }}}{1-p_{e_{\gamma }}}$$
  where $p_{a}$ is the overall agreement probability between experts and $p_{e_{\gamma }}$ is the chance agreement probability (i.e., the probability that the agreement between experts is due to chance), given by
  (2) $$\begin{array}{c} p_{a}=\frac{1}{N}\sum_{i=1}^{N}p_{a_{i}}=\frac{1}{N}\sum_{i=1}^{N}\sum_{j=1}^{M_{i}}\frac{e_{ij}-1}{R(R-1)} \end{array}$$
  (3) $$\begin{array}{c} p_{e_{\gamma }}=\frac{1}{M-1}\sum_{j=1}^{M}\pi_{j}\left(1-\pi_{j}\right) \end{array}$$
  Here, $p_{a_{i}}$ is the agreement probability in situation *i*; *R* is the total number of experts; *N* is the number of finishing situations analysed; $M_{i}=\left|C_{M_{i}}\right|$ is the number of categories (i.e., different options considered by the experts) in situation *i*; $e_{ij}$ is the number of experts that selected the $j^{th}$ option for the $i^{th}$ situation and $\pi_{j}$ is the probability that an expert selects option *j*.
  (4) $$\pi_{j}=\frac{1}{N}\sum_{i=1}^{N}r_{ij}=\frac{1}{N}\sum_{i=1}^{N}\frac{e_{ij}}{R}$$
(c) A multiclass Brier Score (BS) was used to measure the accuracy of the FSV model to predict the choices of the PE coaches. That is, the multiclass BS compares, for each finishing situation, *i*, the fraction, $r_{ij}$, of the PE that chose option *j*, with the probability, $p_{ij}$, assigned by an FSV-based model. Each situation, *i*, contributes to the overall BS with $BS_{i}$, given by
  (5) $$\begin{array}{c} BS_{i}=\frac{1}{2}\sum_{j=1}^{M}{\left(p_{ij}-r_{ij}\right)}^{2} \end{array}$$
  (6) $$\begin{array}{c} BS=\frac{1}{N}\sum_{i=1}^{N}BS_{i} \end{array}$$
  In the FSV-based probabilistic models, each option, *j* in $C_{M}$, is characterized by the stochastic variable $X_{ji}$ in situation *i*. Two different approaches are used:
  **(A)**  **FSV, approach I**, where the probability, $p_{ij}$, that option *j* is selected in situation *i* is given by
    (7) $$p_{ij}=pd_{ij}=P\left(X_{ji}>\max\left\{X_{mi},\ldots ,X_{ni}\right\}\right)$$
    that is, the probability that the value assigned to option *j* is bigger than any of the other options. For option *A* (ball carrier) and *C* to *D* (teammate), $X_{ji}$ is described by a χ distribution with parameter $k_{ji}$ defined by the FSV value corresponding to that option and situation, i.e., $X_{ji}∼\chi \left(FSV_{ji}\right)$. It is important to stress that “Option E" corresponds to not choosing any of the players A to D; thus, there is no FSV for this option. Consequently, the probabilistic model for “Option E” is defined by fitting a skewed normal distribution to the players’ (A to D) FSV values when “Option E” is selected (see Figure A12 in Appendix A).
  **(B)**  **FSV, approach II**, where “Option A” is considered differently from all other options, as it is considered that if the ball carrier (“Option A”) has a “minimum” FSV value, then he/she should shoot. The “minimum” FSV value is described by a normal distribution, $Xt_{Ai}∼N\left(\mu ,\sigma^{2}\right)$, with $\mu ,\sigma^{2}$ fitted to the ball carrier (A) FSV values when option A is selected (see Figure A13 in Appendix A). Consequently, the probability for option A is given by
    (8) $$p_{Ai}=pt_{Ai}+\left(1-pt_{Ai}\right)pd_{Ai}$$
    The first term is associated with the probability of A’s FSV reaching a “minimum” value, i.e., $p_{Ai}=P\left(Xt_{Ai}<FSV_{Ai}\right)$, and the second term is associated to the probability of A’s FSV value being bigger than all other options ($pd_{Ai}$ defined as in Equation (7)). For the remaining options (B to E), $p_{ji}$ is defined by
    (9) $$p_{ji}=\left(1-pt_{Ai}\right)pd_{ji}$$
  In order to assess the “quality” of the two FSV versions, they can be compared with a reference. We used as reference a model where for all situations, the probability that an expert selects option *j* is used, i.e., $p_{ij}=\pi_{j}$ and consequently
  (10) $$BS_{ref}=\frac{1}{2N}\sum_{i=1}^{N}\sum_{j=1}^{M}{\left(\pi_{j}-r_{ij}\right)}^{2}$$

## 3. Results

### 3.1. Comparative Analysis of the Ball Carrier’s Probability to Score

**Figure 5:**
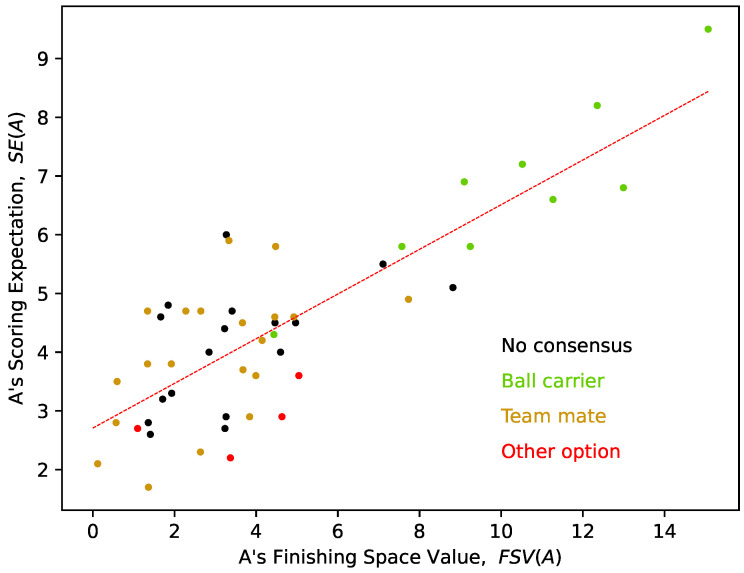
Linear regression between the result of the FSV and the subjective perception of the PE about the “probability to score from a shot” made by player “A” (the ball carrier) in each finishing situation.

Figure 5 presents, for each of the 50 situations considered, the relation between the FSV quantification for player A, FSV(A) (the ball carrier which actually shoots in each situation) and the average results of the PE question about what they consider to be the *probability* to score a goal from that specific shot (on a scale from 0 to 10). The linear regression graph of Figure 5 suggests a “strong association” between the two variables and shows the sensitivity of the FSV model to capture what might be a shot perceived by coaches as having a greater probability of achieving a goal ($R^{2}=0.6706$).

### 3.2. The Coaches’ Opinions

**Figure 6:**
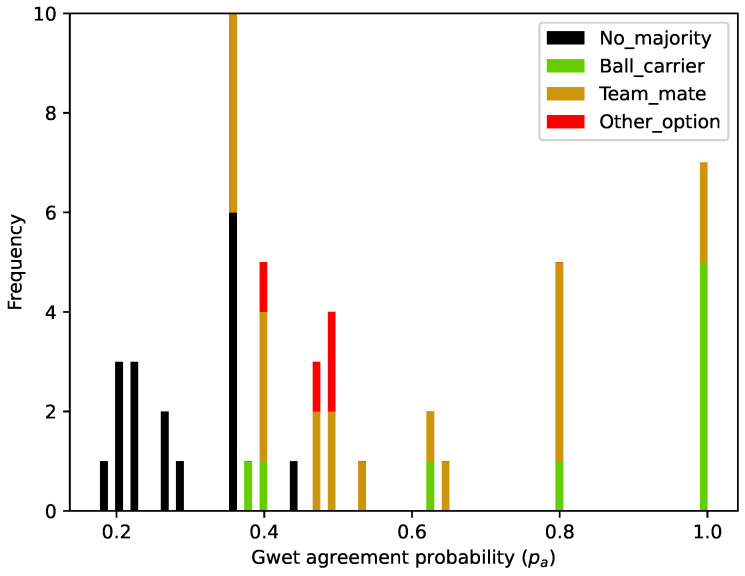
Histogram with the values of the Gwet’s agreement coefficient. It shows the frequency of the situations where the PE did not minimally agree (black: no majority) and the frequency with which the PE produced a tendency in their answers, in the sense that the ”best option” to shoot would be (1) the ball carrier (green: option A); (2) one of his teammates (brown: options B, C or D); or (3) other option (red: E).

The number of coaches that choose each option in each situation is presented in Table A1 of Appendix A, and Figure 6 shows the histogram with the frequency of the respective Gwet’s agreement coefficient.

The total Gwet’s AC1 for this survey was 0.39, demonstrating an agreement among coaches slightly below the “moderate” range (0.40 to 0.60) [48]. We must stress that only in 12 situations (24% of the survey) did the PE coaches score a high agreement (0.80 to 1.00) [48] about what was the “best option” to finish the play. In 18 situations (36% of the survey), the coaches did not express agreement in any sense. The general “fair agreement” (0.21–0.40) among coaches was contrasted with the probabilistic predictions made by the FSV model. This also indicates that the phenomenon itself might be inherently complex, and thus perceived and acted upon in multiple ways.

### 3.3. Comparative Analysis between the PE and the FSV Model

**Figure 7:**
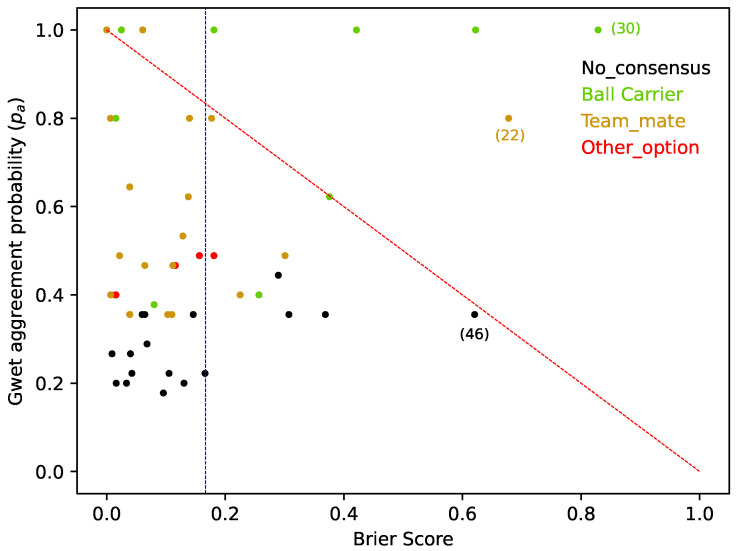
Graph for each situation of the survey, according to Gwet’s agreement probability and the “multiclass BS” for the FSV model (approach I). The vertical blue line indicates the random reference’s BS. (from Equation (10)).

The results of Figure 6 show how coaches *differently* perceive the affordances for players in each finishing situation of the match. However, to compare coaches’ choice probabilities with the predictions of the FSV model, we used a “multiclass BS” (Figure 7).

Figure 7 shows how approach I of the FSV model predicted the PE’s responses, including the finishing situations where the Gwet’s agreement value indicated a low agreement between coaches (see Table A1 and Table A2 of Appendix A). As the BS ranges between 0 (high accuracy) and 1 (low accuracy), the average BS of 0.16 indicates the ability of the FSV model (approach I) to predict the coaches’ answers.

However, Figure 7 also shows the difficulty of the FSV model to adequately predict the coaches’ responses when they predominantly choose option A (i.e., the ball carrier should shoot). In fact, Figure 7 shows (with green bullets) a set of finishing situations where coaches “highly agreed” (Gwet’s AC1=1.0) that the “best option” to the ball carrier was to shoot, but the FSV model (approach I) indicated that other players (B, C or D passing options), with higher values, were better options.

**Figure 8:**
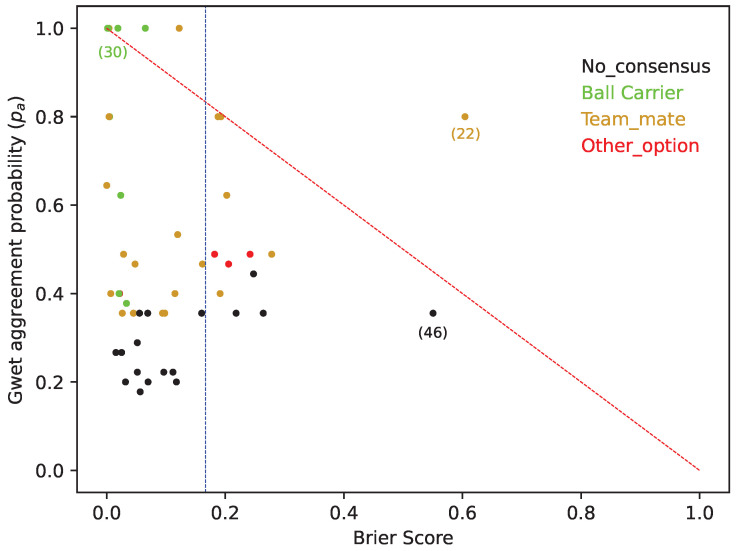
Graph with each finishing situation according to the values of Gwet’s agreement probability and the “multiclass BS” of the FSV model (approach II). The vertical blue line indicates the BS random reference. (from Equation (10)).

Thus, we tested the FSV model (approach II), where the option for a shot by the ball carrier was selected whenever its absolute value was higher than a carefully computed threshold (defined in Figure A13 of Appendix A) *or* when it was higher than that of the other options (B, C, D or E). With approach II, the average BS of the FSV model improves to 0.11, increasing the model accuracy to predict coaches’ choices regardless of their Gwet’s agreement probability (Figure 8). This result means that this FSV model, approach II, was 33% better than the blue line of Figure 8 (the random BS reference, $BS_{ref}$).

Is important to note how two finishing situations (22 and 46 in Figure 8, with results in Table A1 and Table A2) still have a very poor BS, largely above the blue line for the random “BS reference”. These situations indicate the inability of the FSV model (approach II) to correctly predict all of the coaches’ choices.

However, the average BS values of the FSV model (approach I BS=0.16 and approach II BS=0.11) are more accurate in predicting the coaches’ opinions than models only based on players’ locations, i.e., only considering the distance and angle to the goal. In fact, when we consider only the PL component of the FSV model, the BS increased to an average of 0.22 (the lower the better) (Table A3 and in Figure A14 of Appendix A). Very similar results can be observed with the models presented by Pollard and colleagues or by the “Zone” component of Link and colleagues (with an average BS of 0.23 and 0.22, respectively, as expressed in Table A4 and in Figure A15 and Figure A16 of Appendix A).

## 4. Discussion

In the present study, we investigated football players’ decision-making in finishing situations. We hypothesized that players’ decision-making behaviour is based on their perception of the affordances offered by the match [13,19]. Inspired by the “Expected Possession Value” (EPV) model [37,38,39], we built the “Finishing Space Value” (FSV) model, which captures the affordance of shot-on-goal-ability in finishing situations.

The novelty of our study is the simplicity of the parameters that constitute the FSV model. We also used an updated new methodology to validate it. The FSV parameters assess how players perceive the affordances created by information from (a) the distance and angle between each player and the opponent’s goal and (b) the distance between each player and the nearest opponents.

The output of this model is completely distinct from those denominated as expected goal (xG) models [30,31,32,33,34,35,36]. In fact, even if the parameters and computation of each xG model are diverse, the general idea behind it is that the shots’ success ratio provides the “probability” to score from a given location. However, even if this probability can be considered as a general indication of the *reachability* of the opponent’s goal, the output of the FSV model also considers how the “free space” around each player affords them a shot. Therefore, the FSV is a compound quantification of these two affordances to shoot. To test the plausibility of the FSV model, we applied it to finishing situations, and then asked a panel of expert (PE) football coaches their opinion about a sample of those finishing situations. Results showed that the FSV model incorporates information from the affordances for players perceived by the PE [11].

**Figure A11:**
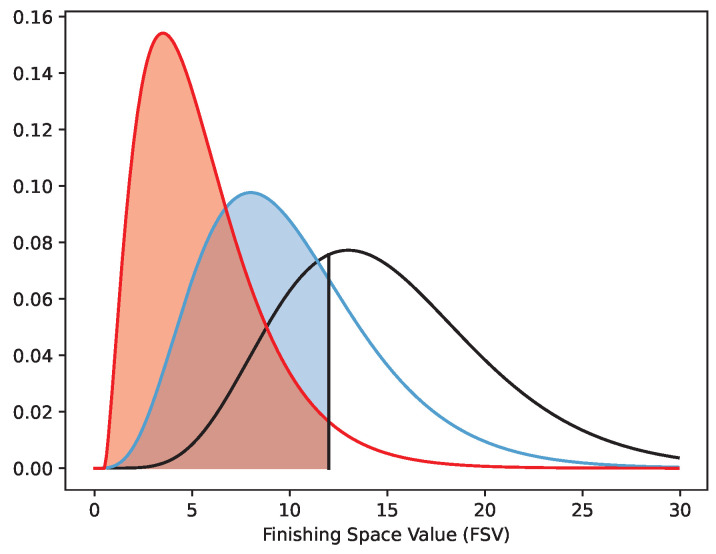
Exemplary graph of how the FSV of different players was compared. In this case, the probability that player A (black line) has a higher FSV than B (blue line) or C (red line) is given by the respective shaded zones.

**Figure A12:**
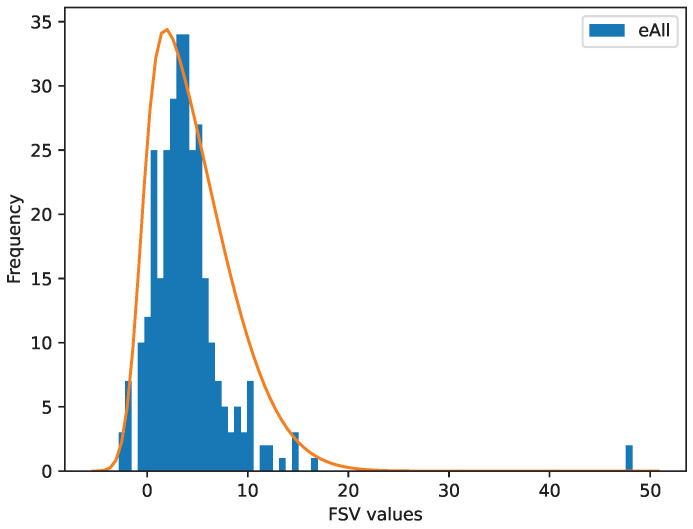
Histogram with the values of the FSV of each player (A, B, C and D) when each coach chose “E” as the “best option” (continue to play, and not shoot or pass to shoot).

**Figure A13:**
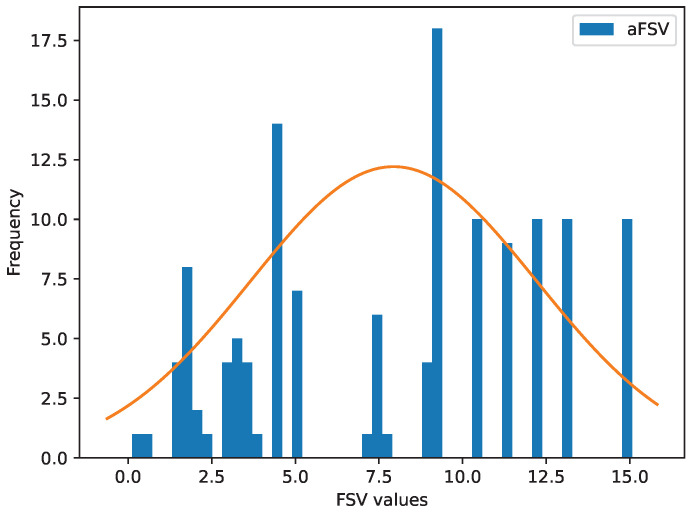
Histogram with the frequency of the FSV of the ball carrier (player A) when each coach chose him as the “best option”.

Importantly, the PE and the FSV model are highly correlated in their ability to predict when a shot will be made ($R^{2}=0.6706$). However, in most of the finishing situations presented to the PE, there was no unanimity in the answers of the coaches. This is demonstrated by a general agreement between coaches that results in a Gwet’s AC1 of only 0.39. This indicates that finishing situations as a whole are inherently complex, and thus perceived and acted upon in multiple ways. Coaches perceive the affordances for athletes in multiple ways, maybe as diverse as how athletes perceive the affordances to shooting the ball themselves. Affordances are perceived according to the skills and characteristics of an athlete as well as according to the specificity of the task [23]. So, if the phenomenon is well captured by the FSV model, it should also express such diversity of how the phenomenon can be perceived and acted upon. Importantly, the results of the multiclass BS that measured the accuracy of the probabilistic predictions made by the FSV model achieved a value of 0.16. This result, obtained with approach I, was based on a simple comparison of each option of the FSV (see Figure 7 and Figure A11). Nevertheless, in this approach, it was demonstrated (see for example, the situation (30) in Figure 7) that football coaches considered that when the ball carrier is “sufficiently” well located and has enough space to score, they should shoot [49], even when there are other teammates in a slightly better contextual position (as the FSV model captured). With approach II, it was assumed that the FSV model will always choose option A (the ball carrier should shoot) when its value is bigger than a threshold (given by the data expressed in Figure A13). Interestingly, for this second approach, the BS achieve a value of 0.11 (Figure 8), demonstrating how coaches can be sensitive to that kind of perceived thresholds that differentiate the ball carrier from all other teammates [46,50,51]. To quote Carlos Queiroz, “The worst mistake we can make in a finishing situation is to not take the shot when we are close enough to the opposing goal and with space to do it” [50].

**Figure A14:**
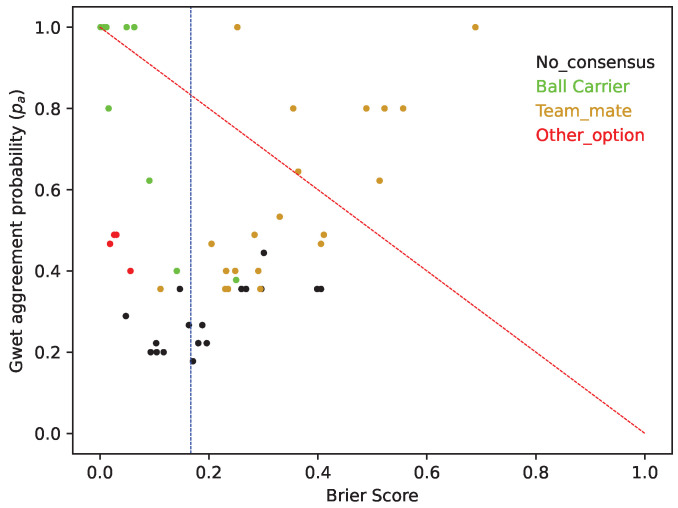
Graph showing, for each situation of the survey, the comparison between the values of Gwet’s agreement probability and the “multiclass BS” that measures the accuracy of probabilistic predictions if the FSV model only has the PL (distance and angle to the opponent’s goal). The vertical blue line indicates a BS random reference.

**Figure A15:**
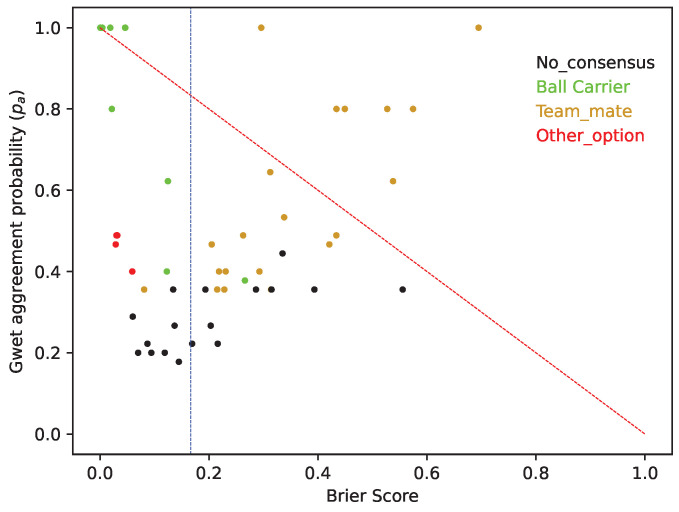
Graph showing, for each situation of the survey, the comparison between the values of Gwet’s agreement probability and the “multiclass BS” that measures the accuracy of probabilistic predictions with the model proposed by Pollard and colleagues [28]. The vertical blue line indicates a BS random reference.

**Figure A16:**
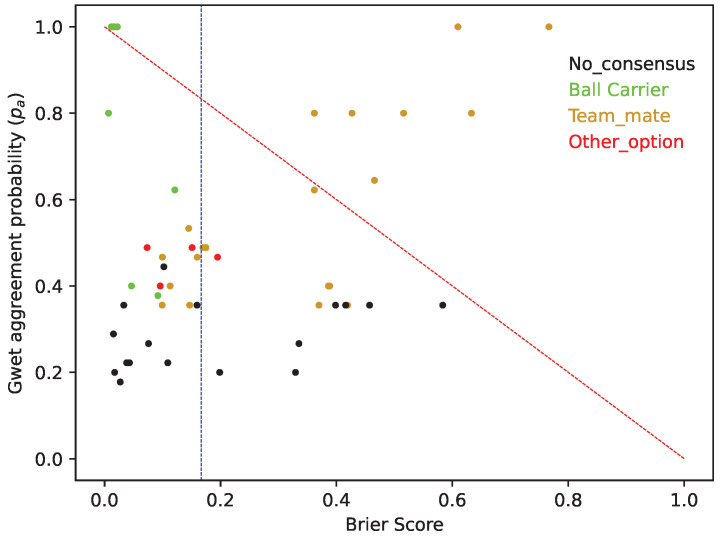
Graph showing, for each situation of the survey, the comparison between the values of Gwet’s agreement probability and the “multiclass BS” that measures the accuracy of probabilistic predictions with the “Zone” component of the “Dangerousity” model proposed by Link and colleagues [29]. The vertical blue line indicates a BS random reference.

Finally, we conducted a comparison of the BS achieved by the FSV model in the two computational approaches (0.16 and 0.11), with the situation where only the component PL was considered (see Figure A14). In this case, the BS was the worst (0.22) and very similar to the one obtained when we applied the functions proposed by Pollard and colleagues [28] (see Figure A15) or the one originated by the “Zone” component of the model proposed by Link and colleagues [29] (see Figure A16). These results demonstrated how the contextual information about the “free space” around each player contributes to increasing the accuracy of the model and is relevant for the understanding of dynamic ecologically situated decision-making behaviour in finishing situations in football.

## 5. Conclusions

The Finishing Space Value (FSV) model demonstrates its ability to capture the affordances that can guide players during their decisions and actions in finishing situations. In fact, when compared with expert football coaches, the FSV seems to be able to quantify the opportunities for shooting in the game’s landscape in a very similar way, as demonstrated by the Brier Score (BS), which contrasts the coaches’ opinions with the FSV results. Importantly, the BS of the FSV model that includes the relativization of the “free space” around each player is better than the BS of other models where this relativization is not present. This shows how the majority of expected goal (xG) models fail short in capturing the probabilities to score from some pitch locations if they do not include the assessment of the game’s spatial landscape and its affordances to shoot, which derive from the dynamics of the two teams in confrontation.

Despite the encouraging results of this study, we are aware that the model needs to be further tested with larger and more diverse data from matches. The phenomenon is inherently complex as it was also expressed by the diversity of opinions of expert football coaches about a given situation. The perception of affordances for players expressed by coaches is influenced by their unique paths in football, embedded in their sociocultural history and forms of life [52]. For example, the PE of this study, although performing in high-level football worldwide, were all Portuguese [53]. Likewise, an important line of future research could focus on the opinion of high-level professional players.

The future testing of the FSV model should also include more diverse positional data (e.g., from different competitions) to be improved. This will also contribute to overcoming the limitations of the use of simple VDs as a proxy to “free space” around each player [54]. However, the substitution of VDs by more complex models of “dominant regions” [55] that include players’ trajectories and speeds is not an easy path [56]. It implies transdisciplinary research about sport behaviour, needing to join football players and coaches’ experiential knowledge with sports scientists, sports psychologists and data scientists [57].

The FSV model might contribute to several practical applications:

(a) For scouting, the quantification of the players’ FSV can support recruitment processes. Notably, in high-performance contexts, increasingly supported by data [58], the FSV can allow us to differentiate between “the efficiency of the shooter” from the “difficulty of the shot” ([59], p. 22).
(b) For match analysis, identifying [60] the game moments when a given player or team achieves higher FSV values can contribute to improve coaches’ decisions about the game [61].
(c) For practice, applying FSV to the analysis of performance in *representative* practice tasks might be used to inform how such practice transfers to performance on the match [62]. Thus, coaches can design and better manipulate practice task constraints [63].

## Figures and Tables

**Table 1:** Ball carrier’s *VAe* in function of the *distance* to the centre of opponent’s goal line (*x*) for each *region* of the EPS.

EPS Region	Formulas for the Ball Carrier (BC)
INS	$=-0.0035x^{3}+0.2125x^{2}-2.0532x+24.5210$
OUT_F	$=-0.0630x^{2}+5.3921x+6.4489$
OUT_S	$=0.0181x^{3}-1.9781x^{2}+61.8270x-335.7300$
OUT_S_F	$=0.0262x^{3}-2.1278x^{2}+47.7160x+11.4520$

**Table 2:** Teammates’ of the ball carrier without the ball *VAe* in function of the *distance* to the centre of opponent’s goal line (*x*) for each *region* of the EPS.

EPS Region	Formulas for the Ball Carrier’s Teammates (NB)
INS	$=-0.0057x^{3}+0.3018x^{2}-1.5952x+14.1360$
OUT_F	$=-0.1019x^{2}+8.1389x-9.8798$
OUT_S	$=0.0118x^{3}-1.7250x^{2}+61.1630x-201.0100$
OUT_S_F	$=0.0361x^{3}-3.0145x^{2}+77.3510x-136.5000$

**Figure A1:**
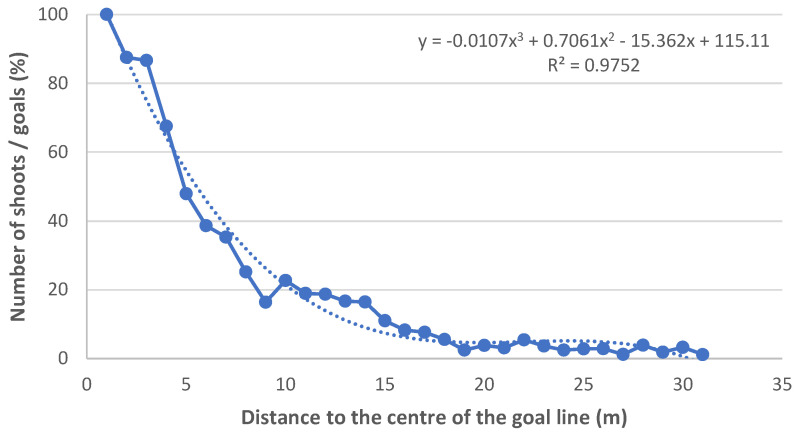
PL and how the distance to the goal influences scoring probability percentage.

**Figure A2:**
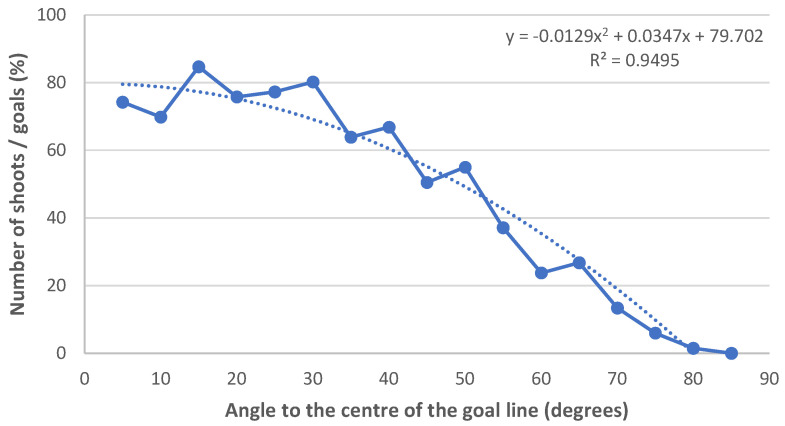
PL and how the angle to the goal influences scoring probability percentage.

**Figure A6:**
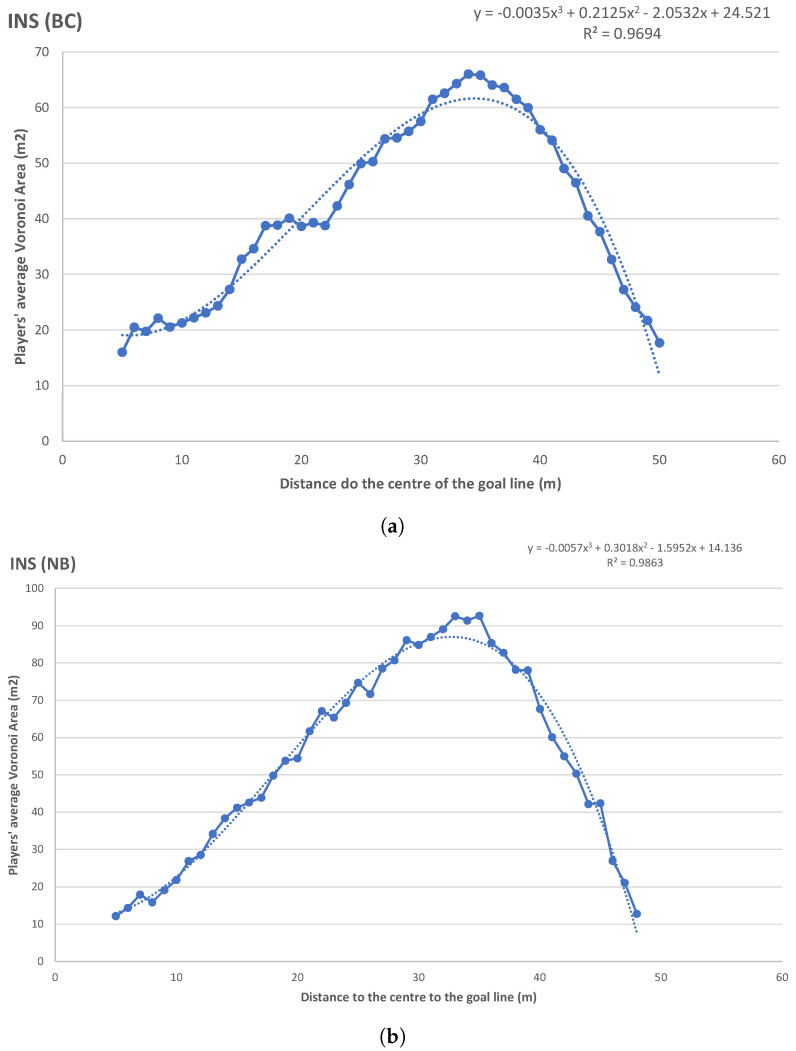
Variation in the average values of the VA of players who are with (BC) and without the ball (NB), in the inside (INS) zones of the EPS. (**a**) VAe for the ball carrier (BC) INSIDE the EPS; (**b**) VAe for players without the ball (NB) INSIDE the EPS.

**Figure A7:**
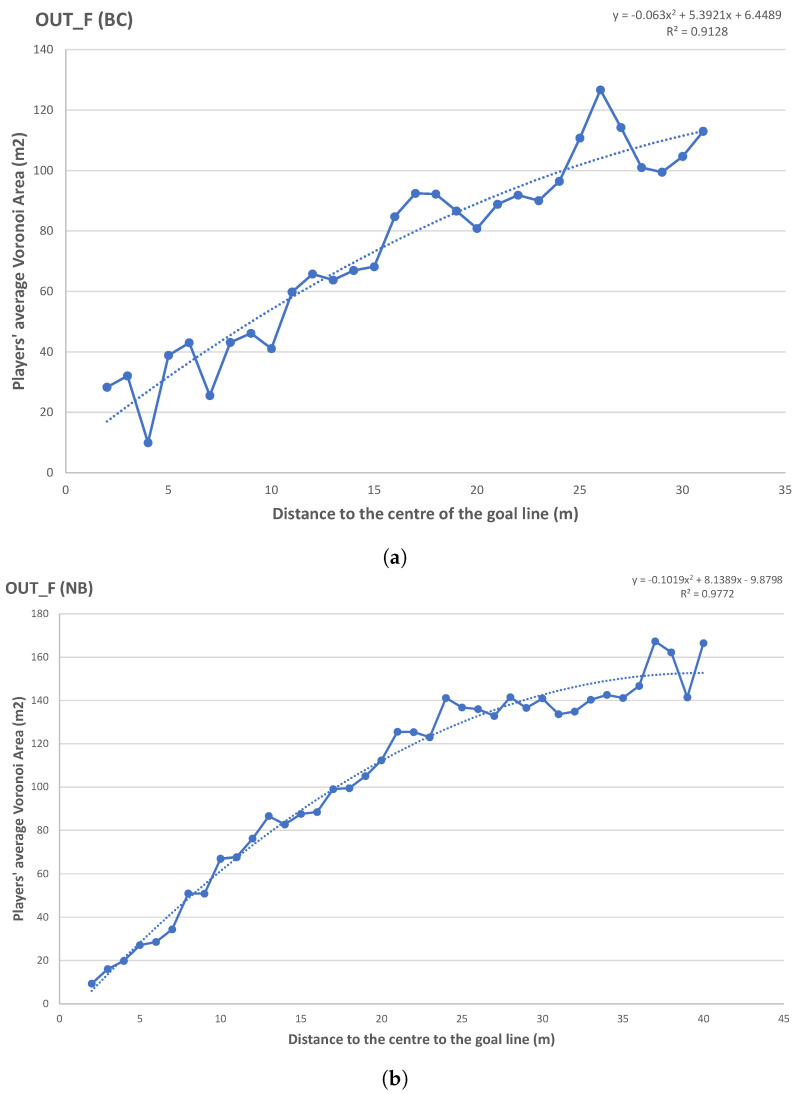
Variation of the average values of the VA of players who are with (BC) and without the ball (NB), in the Outside Frontal region (OUT_F) of the EPS. (**a**) VAe for the ball carrier (BC) in the Outside Frontal region (OUT_F) of the EPS; (**b**) VAe for players without the ball (NB) in the Outside Frontal region (OUT_F) of the EPS.

**Figure A8:**
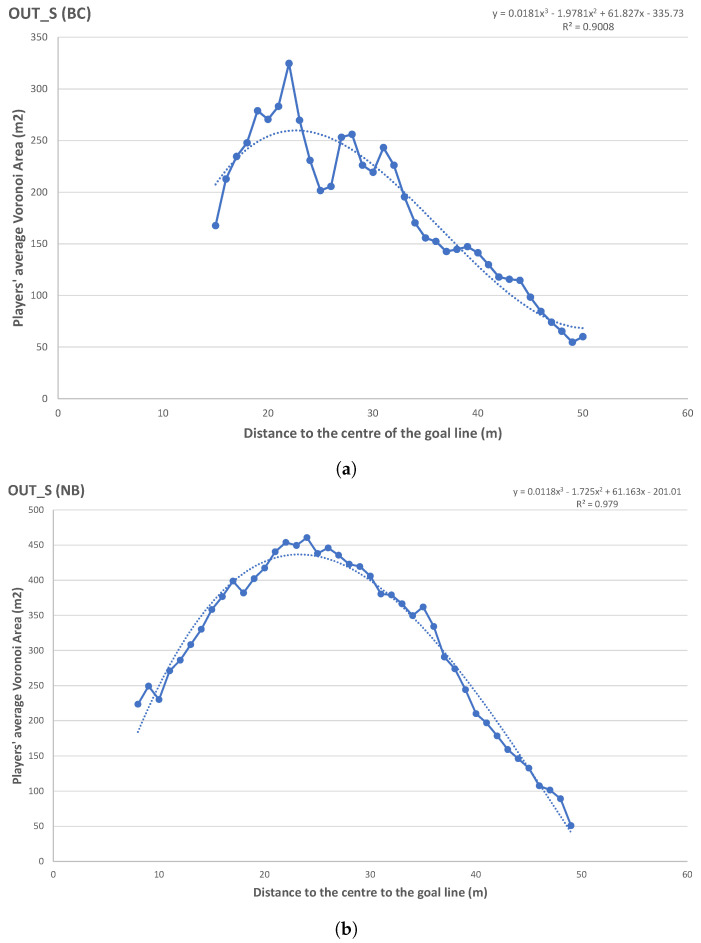
Variation in the average values of the VA of players who are with (BC) and without the ball (NB), in the Outside Side regions (OUT_S) of the EPS. (**a**) VAe for the ball carrier (BC) in the Outside Side region (OUT_S) of the EPS; (**b**) VAe for players without the ball (NB) in the Outside Side region (OUT_S) of the EPS.

**Figure A9:**
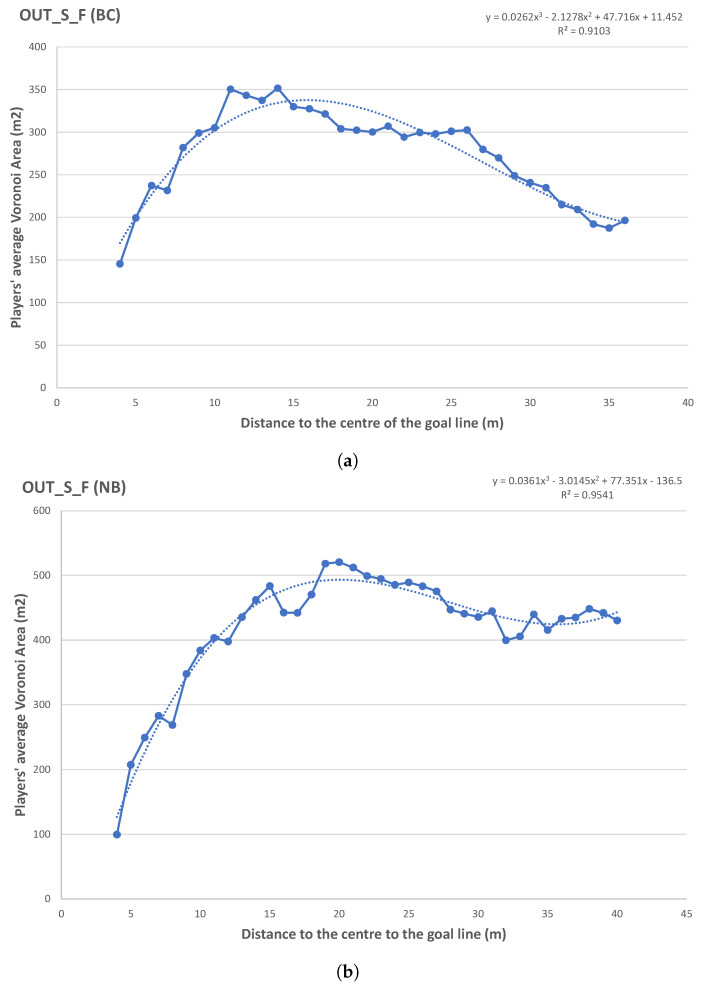
Variation in the average values of the VA of players who are with (BC) and without the ball (NB), in the Outside side–frontal regions (OUT_S_F) of the EPS. (**a**) VAe for the ball carrier (BC) in the Outside side–frontal region (OUT_S) of the EPS; (**b**) VAe for players without the ball (NB) in the Outside side–frontal region (OUT_S_F) of the EPS.

**Figure A10:**
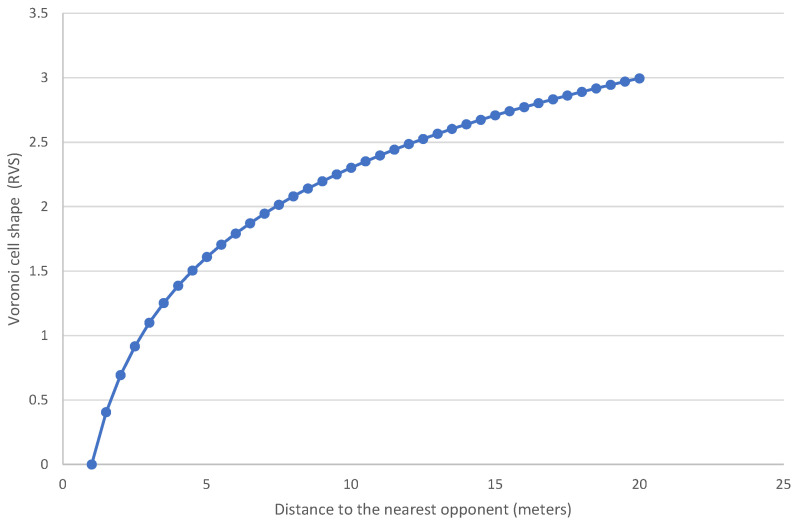
Graph with the function of the natural logarithm (LN) that is introduced in the FSV model to capture the shape of the Voronoi cell, through the distance to the nearest opponent (DO).

## Data Availability

Third party data. Restrictions apply to the availability of these data. Data were obtained from STATS^©^ company and are available from STATS^©^ with their permission.

## Abbreviations

The following abbreviations are used in this manuscript:

BS	Brier Score
EPS	Effective Playing Space
EPV	Expected Possession Value
FSV	Finishing Space Value
PE	Panel of Expert (coaches)
PL	Player Location
RVA	Relative Voronoi Area
RVP	Relative Voronoi Position
VA	Voronoi Area
VAe	Expected Voronoi Area
VC	Voronoi Cell
VD	Voronoi Diagram
xG	Expected Goal

## Appendix A. Additional Figures and Tables

**Table A1:** The table presents the results of the number of coaches that choose each possibility (A, B, C, D or E) in each one of the 50 finishing situations of the survey answered by the PE choices. The agreement between the coaches of the PE is calculated by the Gwet’s AC1. In the following columns, the results of the FSV for each player in each situation (A, B, C and D) is shown. Finally, the last two columns present the BSs that compare the probability of choosing one given “best option” (A, B, C, D or E) in the two approaches: by the subjective answers of a PE coach and by the two versions of the FSV quantification model.

Situation	PE Choices						FSV Values (A.U.)				BS	
	A	B	C	D	E	Gwet	A	B	C	D	Model I	Model II
01	4	2	0	1	3	**0.22**	2.85	2.74	0.00	6.98	0.17	0.11
02	0	3	1	4	2	**0.22**	3.26	9.07	5.07	6.41	0.04	0.05
03	6	0	0	2	2	**0.38**	4.43	3.67	0.45	2.85	0.08	0.03
04	10	0	0		0	**1.00**	10.52	0,60	0.00		0.02	0.00
05	2	1	2	4	1	**0.18**	4.97	12.03	13.73	10.47	0.10	0.06
06	1	6	3	0	0	**0.40**	7.73	21.08	15.74	3.24	0.01	0.12
07	1	5	0	2	2	**0.27**	4.46	14.87	4.87	9.84	0.04	0.03
08	3	7	0		0	**0.53**	4.48	6.98	6.32		0.13	0.12
09	0	3	1	0	6	**0.40**	1.10	1.98	0.06	0.91	0.02	0.02
10	0	2	0	7	1	**0.49**	2.64	3.15	5.45	11.40	0.02	0.03
11	1	6	2	0	1	**0.36**	3.84	9.39	0.00	5.87	0.04	0.05
12	1	1	6	0	2	**0.36**	0.12	5.07	48.22	6.03	0.11	0.09
13	3	1	0	1	5	**0.29**	1.84	3.95	3.29	3.60	0.07	0.05
14	0	4	5	0	1	**0.36**	3.24	4.71	10.19	3.65	0.06	0.07
15	4	1	5		0	**0.36**	3.27	12.28	7.65		0.31	0.22
16	2	0	7	0	1	**0.49**	1.34	11.44	8.23	8.38	0.30	0.28
17	0	9	0	0	1	**0.80**	2.64	8.73	8.22	2.25	0.18	0.19
18	1	2	6	1	0	**0.36**	1.34	5.22	15.16	8.39	0.04	0.03
19	0	10	0		0	**1.00**	3.99	11.57	6.57		0.06	0.12
20	0	1	2	0	7	**0.49**	3.37	0.00	4.47	5.33	0.13	0.18
21	1	0	6	3	0	**0.40**	2.28	2.65	14.30	21.81	0.23	0.19
22	0	1	9	0	0	**0.80**	3.33	23.92	9.69	13.77	0.68	0.60
23	0	9	0	0	1	**0.80**	3.66	7.14	5.35	1.44	0.14	0.19
24	9	0	1	0	0	**0.80**	11.27	0.00	4.65	3.08	0.02	0.01
25	5	5	0	0	0	**0.44**	1.67	2.86	4.21	1.78	0.29	0.25
26	4	0	5	0	1	**0.36**	3.41	1.80	10.18	14.74	0.37	0.26
27	4	0	6		0	**0.47**	4.92	4.56	7.45		0.06	0.05
28	1	1	0	1	7	**0.47**	5.05	0.46	2.45	2.36	0.12	0.21
29	2	2	0	1	7	**0.49**	4.63	3.82	5.49	0.00	0.18	0.24
30	10	0	0	0	0	**1.00**	12.35	9.65	26.99	16.53	0.83	0.02
31	6	0	0	3	1	**0.40**	7.57	12.27	1.86	16.57	0.26	0.02
32	1	4	1	1	3	**0.20**	3.23	7.74	6.83	3.27	0.03	0.03
33	0	10	0	0	0	**1.00**	1.36	69.82	22.81	7.19	0.00	0.00
34	0	0	4	3	3	**0.27**	1.71	3.37	6.36	5.03	0.01	0.02
35	0	1	5	0	4	**0.36**	1.36	3.23	10.01	3.81	0.06	0.06
36	1	0	1	6	2	**0.36**	0.57	4.23	0.45	4.45	0.10	0.10
37	8	0	1	1	0	**0.62**	9.24	8.46	5.72	14.77	0.38	0.02
38	10	0	0	0	0	**1.00**	9.10	0.00	11.37	8.80	0.42	0.07
39	2	2	0	4	2	**0.20**	4.60	6.34	5.82	0.02	0.13	0.12
40	1	4	5	0	0	**0.36**	7.10	15.72	8.50	2.54	0.15	0.16
41	0	1	6	0	3	**0.40**	0.60	0.00	6.42	0.64	0.01	0.01
42	4	3	1	1	1	**0.20**	8.82	8.96	2.00	7.72	0.02	0.07
43	1	4	2	0	3	**0.22**	1.41	2.00	5.61	3.97	0.11	0.10
44	2	8	0	0	0	**0.64**	4.46	31.12	9.02	1.81	0.04	0.00
45	10	0	0	0	0	**1.00**	15.07	8.59	21.95	1.03	0.62	0.00
46	1	5	4	0	0	**0.36**	1.93	8.84	0.00	20.52	0.62	0.55
47	10	0	0	0	0	**1.00**	12.99	3.23	10.11	10.13	0.18	0.00
48	0	8	1		1	**0.62**	4.15	5.35	2.92		0.14	0.20
49	1	9	0	0	0	**0.80**	1.92	16.33	5.29	5.22	0.01	0.00
50	0	0	6	0	0	**0.47**	3.68	2.11	3.53	2.51	0.11	0.16
**Gwet AC1**						**0.39**		**BS**			0.16	0.11

**Table A2:** The table specifies the results of the probabilities of the FSV quantification model (versions I and II) to choose one “best option” in each finishing situation. In each FSV model (I and II), the last column presents the BS when these probabilities were compared with the probabilities calculated from the PE coaches’ subjective answers.

Situation	Probabilities of FSV Model (I)						Probabilities of FSV Model (II)					
	A	B	C	D	E	BS(I)	A	B	C	D	E	BS(II)
01	0.09	0.08	0.00	0.57	0.27	0.17	0.19	0.07	0.00	0.50	0.24	0.11
02	0.04	0.51	0.11	0.21	0.13	0.04	0.17	0.44	0.10	0.18	0.11	0.05
03	0.30	0.21	0.01	0.13	0.35	0.08	0.45	0.17	0.01	0.10	0.28	0.03
04	0.84	0.00	0.00	0.00	0.16	0.02	0.96	0.00	0.00	0.00	0.04	0.00
05	0.02	0.30	0.46	0.19	0.03	0.10	0.26	0.23	0.35	0.15	0.02	0.06
06	0.02	0.72	0.25	0.00	0.01	0.01	0.49	0.38	0.13	0.00	0.00	0.12
07	0.02	0.72	0.02	0.21	0.04	0.04	0.22	0.57	0.02	0.16	0.03	0.03
08	0.13	0.38	0.30	0.00	0.19	0.13	0.31	0.30	0.24	0.00	0.15	0.12
09	0.09	0.21	0.00	0.07	0.63	0.02	0.14	0.20	0.00	0.07	0.59	0.02
10	0.02	0.03	0.11	0.74	0.11	0.02	0.12	0.02	0.10	0.66	0.09	0.03
11	0.07	0.60	0.00	0.19	0.15	0.04	0.22	0.50	0.00	0.15	0.12	0.05
12	0.00	0.00	1.00	0.00	0.00	0.11	0.03	0.00	0.97	0.00	0.00	0.09
13	0.06	0.23	0.16	0.20	0.35	0.07	0.13	0.22	0.15	0.18	0.32	0.05
14	0.04	0.10	0.68	0.05	0.13	0.06	0.17	0.08	0.59	0.05	0.12	0.07
15	0.02	0.71	0.19	0.00	0.08	0.31	0.16	0.61	0.17	0.00	0.07	0.22
16	0.00	0.52	0.20	0.21	0.07	0.30	0.06	0.49	0.19	0.20	0.06	0.28
17	0.02	0.45	0.38	0.02	0.13	0.18	0.13	0.40	0.34	0.01	0.12	0.19
18	0.00	0.03	0.79	0.13	0.04	0.04	0.06	0.03	0.74	0.13	0.04	0.03
19	0.04	0.71	0.16	0.00	0.10	0.06	0.21	0.58	0.13	0.00	0.08	0.12
20	0.13	0.00	0.24	0.34	0.29	0.16	0.25	0.00	0.20	0.29	0.25	0.18
21	0.00	0.00	0.18	0.81	0.01	0.23	0.09	0.00	0.16	0.74	0.01	0.19
22	0.00	0.86	0.03	0.11	0.00	0.68	0.14	0.74	0.02	0.09	0.00	0.60
23	0.10	0.45	0.23	0.01	0.21	0.14	0.24	0.38	0.19	0.01	0.18	0.19
24	0.77	0.00	0.08	0.03	0.12	0.02	0.95	0.00	0.02	0.01	0.03	0.01
25	0.06	0.15	0.32	0.07	0.40	0.29	0.13	0.14	0.30	0.06	0.37	0.25
26	0.01	0.00	0.24	0.71	0.04	0.37	0.15	0.00	0.20	0.61	0.03	0.26
27	0.18	0.15	0.47	0.00	0.20	0.06	0.38	0.11	0.36	0.00	0.15	0.05
28	0.42	0.01	0.10	0.10	0.37	0.12	0.57	0.01	0.08	0.07	0.28	0.21
29	0.23	0.15	0.34	0.00	0.27	0.18	0.40	0.12	0.26	0.00	0.21	0.24
30	0.03	0.01	0.84	0.12	0.00	0.83	0.85	0.00	0.13	0.02	0.00	0.02
31	0.05	0.26	0.00	0.67	0.02	0.26	0.49	0.14	0.00	0.36	0.01	0.02
32	0.05	0.42	0.31	0.05	0.17	0.03	0.18	0.36	0.27	0.05	0.14	0.03
33	0.00	1.00	0.00	0.00	0.00	0.00	0.06	0.94	0.00	0.00	0.00	0.00
34	0.03	0.10	0.40	0.24	0.24	0.01	0.10	0.09	0.37	0.22	0.22	0.02
35	0.01	0.05	0.72	0.07	0.15	0.06	0.07	0.04	0.68	0.07	0.14	0.06
36	0.01	0.29	0.01	0.32	0.37	0.10	0.05	0.28	0.01	0.31	0.35	0.10
37	0.15	0.12	0.03	0.66	0.03	0.38	0.68	0.04	0.01	0.25	0.01	0.02
38	0.25	0.00	0.47	0.22	0.06	0.42	0.70	0.00	0.18	0.09	0.02	0.07
39	0.16	0.34	0.28	0.00	0.21	0.13	0.35	0.27	0.22	0.00	0.17	0.12
40	0.07	0.78	0.12	0.00	0.03	0.15	0.46	0.45	0.07	0.00	0.02	0.16
41	0.01	0.00	0.64	0.01	0.34	0.01	0.05	0.00	0.61	0.01	0.33	0.01
42	0.33	0.34	0.01	0.23	0.09	0.02	0.72	0.14	0.00	0.10	0.04	0.07
43	0.03	0.05	0.41	0.20	0.31	0.11	0.09	0.05	0.39	0.19	0.29	0.10
44	0.00	0.99	0.00	0.00	0.00	0.04	0.21	0.79	0.00	0.00	0.00	0.00
45	0.20	0.02	0.78	0.00	0.00	0.62	0.96	0.00	0.04	0.00	0.00	0.00
46	0.00	0.05	0.00	0.93	0.01	0.62	0.08	0.05	0.00	0.86	0.01	0.55
47	0.50	0.01	0.23	0.23	0.04	0.18	0.94	0.00	0.03	0.03	0.00	0.00
48	0.22	0.37	0.11	0.00	0.31	0.14	0.36	0.30	0.09	0.00	0.25	0.20
49	0.00	0.90	0.03	0.03	0.04	0.01	0.08	0.83	0.03	0.03	0.03	0.00
50	0.23	0.08	0.21	0.11	0.37	0.11	0.35	0.07	0.18	0.09	0.31	0.16
**Mean**						**0.16**	**Mean**					**0.11**

**Table A3:** The table presents the results of the probabilities of a quantification model that only includes the PL (distance and angle to the opponent’s goal) to choose one “best option” in each finishing situation. The last column presents the BS of this model (BS PL) when these probabilities were compared with the probabilities calculated from the PE coaches’ subjective answers.

Situation	Probabilities of PL Model					
	A	B	C	D	E	BS(PL)
01	0.10	0.11	0.14	0.05	0.59	0.10
02	0.07	0.05	0.19	0.07	0.62	0.18
03	0.15	0.04	0.05	0.03	0.72	0.25
04	0.89	0.01	0.00	0.00	0.10	0.01
05	0.17	0.09	0.13	0.05	0.56	0.17
06	0.27	0.11	0.08	0.03	0.51	0.29
07	0.29	0.09	0.03	0.07	0.52	0.16
08	0.47	0.04	0.04	0.00	0.45	0.33
09	0.05	0.02	0.08	0.07	0.77	0.06
10	0.14	0.02	0.07	0.07	0.70	0.41
11	0.37	0.05	0.15	0.04	0.38	0.23
12	0.03	0.26	0.24	0.04	0.44	0.11
13	0.17	0.02	0.03	0.03	0.75	0.05
14	0.32	0.07	0.05	0.04	0.52	0.30
15	0.21	0.12	0.09	0.00	0.58	0.27
16	0.30	0.09	0.07	0.04	0.50	0.28
17	0.35	0.05	0.04	0.01	0.54	0.52
18	0.28	0.14	0.09	0.09	0.41	0.24
19	0.23	0.06	0.04	0.00	0.67	0.69
20	0.16	0.02	0.04	0.04	0.74	0.03
21	0.17	0.02	0.07	0.27	0.46	0.25
22	0.30	0.14	0.18	0.07	0.30	0.35
23	0.29	0.03	0.06	0.01	0.62	0.56
24	0.81	0.04	0.02	0.01	0.12	0.02
25	0.28	0.06	0.05	0.01	0.60	0.30
26	0.19	0.01	0.06	0.11	0.62	0.26
27	0.19	0.07	0.05	0.00	0.68	0.41
28	0.18	0.07	0.11	0.06	0.58	0.02
29	0.10	0.02	0.09	0.12	0.66	0.03
30	0.70	0.03	0.12	0.02	0.13	0.06
31	0.27	0.05	0.17	0.09	0.41	0.14
32	0.30	0.04	0.06	0.05	0.55	0.12
33	0.04	0.39	0.10	0.20	0.28	0.25
34	0.23	0.01	0.04	0.06	0.66	0.19
35	0.08	0.06	0.09	0.03	0.74	0.15
36	0.17	0.03	0.07	0.03	0.71	0.29
37	0.54	0.06	0.03	0.05	0.32	0.09
38	0.90	0.03	0.01	0.00	0.05	0.01
39	0.17	0.05	0.08	0.16	0.55	0.10
40	0.72	0.03	0.02	0.01	0.21	0.40
41	0.08	0.05	0.07	0.08	0.71	0.23
42	0.41	0.03	0.07	0.05	0.43	0.09
43	0.08	0.05	0.05	0.03	0.79	0.20
44	0.22	0.14	0.07	0.03	0.54	0.36
45	0.96	0.00	0.01	0.00	0.03	0.00
46	0.10	0.06	0.00	0.18	0.65	0.41
47	0.73	0.01	0.10	0.03	0.13	0.05
48	0.19	0.02	0.06	0.00	0.72	0.51
49	0.14	0.13	0.05	0.07	0.61	0.49
50	0.21	0.02	0.07	0.01	0.69	0.20
**Mean**						**0.22**

**Table A4:** The table specifies the results of the probabilities of the quantification models already presented by Pollard and colleagues [28] and Link and colleagues [29] (in this case, only to its component “Zone”). The last column of each model presents the BS, when these probabilities were compared with the probabilities calculated from the PE coaches’ subjective answers.

Situation	Probabilities of the Model: Pollard et al. [28]						Probabilities of the Model: Link et al. [29]					
	A	B	C	D	E	BS	A	B	C	D	E	BS
01	0.11	0.15	0.19	0.04	0.51	0.09	0.08	0.29	0.31	0.11	0.20	0.11
02	0.07	0.04	0.23	0.07	0.58	0.17	0.08	0.14	0.30	0.28	0.19	0.04
03	0.15	0.03	0.05	0.02	0.75	0.27	0.25	0.13	0.17	0.13	0.31	0.09
04	0.85	0.02	0.00	0.00	0.13	0.02	0.85	0.06	0.00	0.00	0.08	0.02
05	0.16	0.11	0.18	0.05	0.50	0.14	0.20	0.22	0.24	0.21	0.12	0.03
06	0.52	0.09	0.07	0.01	0.31	0.29	0.77	0.08	0.08	0.03	0.04	0.39
07	0.27	0.12	0.03	0.09	0.49	0.14	0.77	0.08	0.03	0.08	0.04	0.34
08	0.41	0.05	0.04	0.00	0.49	0.34	0.41	0.24	0.14	0.00	0.21	0.15
09	0.06	0.01	0.09	0.08	0.76	0.06	0.03	0.13	0.26	0.26	0.33	0.10
10	0.08	0.01	0.08	0.07	0.75	0.43	0.05	0.15	0.22	0.22	0.36	0.17
11	0.42	0.05	0.16	0.04	0.32	0.23	0.81	0.05	0.06	0.05	0.02	0.42
12	0.10	0.29	0.27	0.03	0.32	0.08	0.20	0.25	0.25	0.19	0.12	0.10
13	0.16	0.01	0.02	0.01	0.79	0.06	0.15	0.16	0.07	0.09	0.53	0.02
14	0.30	0.09	0.05	0.03	0.53	0.29	0.78	0.08	0.05	0.05	0.05	0.46
15	0.30	0.14	0.10	0.00	0.46	0.19	0.78	0.09	0.08	0.00	0.05	0.16
16	0.25	0.13	0.10	0.03	0.48	0.26	0.38	0.19	0.19	0.12	0.11	0.17
17	0.32	0.05	0.04	0.00	0.58	0.53	0.49	0.14	0.15	0.01	0.21	0.43
18	0.39	0.15	0.09	0.09	0.28	0.22	0.76	0.08	0.07	0.07	0.03	0.37
19	0.23	0.06	0.03	0.00	0.67	0.69	0.80	0.06	0.05	0.00	0.09	0.77
20	0.08	0.01	0.04	0.03	0.84	0.03	0.05	0.14	0.17	0.24	0.41	0.07
21	0.24	0.01	0.08	0.27	0.39	0.22	0.77	0.03	0.07	0.09	0.04	0.39
22	0.53	0.12	0.13	0.06	0.16	0.45	0.76	0.07	0.07	0.06	0.03	0.63
23	0.25	0.02	0.07	0.00	0.66	0.57	0.37	0.17	0.17	0.01	0.28	0.36
24	0.77	0.07	0.03	0.00	0.13	0.02	0.82	0.07	0.06	0.01	0.04	0.01
25	0.23	0.07	0.04	0.00	0.65	0.34	0.35	0.19	0.18	0.04	0.24	0.10
26	0.04	0.00	0.00	0.00	0.96	0.56	0.01	0.01	0.01	0.01	0.98	0.58
27	0.17	0.08	0.05	0.00	0.69	0.42	0.25	0.23	0.21	0.00	0.31	0.16
28	0.17	0.09	0.15	0.05	0.54	0.03	0.22	0.25	0.25	0.13	0.16	0.19
29	0.09	0.01	0.11	0.16	0.63	0.03	0.04	0.12	0.23	0.34	0.27	0.15
30	0.74	0.04	0.11	0.02	0.09	0.05	0.86	0.05	0.05	0.03	0.02	0.01
31	0.28	0.05	0.20	0.12	0.35	0.12	0.77	0.05	0.08	0.07	0.04	0.05
32	0.29	0.03	0.06	0.06	0.56	0.12	0.78	0.03	0.05	0.09	0.05	0.33
33	0.17	0.32	0.11	0.22	0.19	0.30	0.63	0.11	0.10	0.11	0.04	0.61
34	0.20	0.00	0.03	0.06	0.71	0.20	0.27	0.03	0.15	0.19	0.37	0.08
35	0.09	0.05	0.11	0.02	0.73	0.13	0.09	0.17	0.34	0.12	0.28	0.03
36	0.16	0.01	0.08	0.02	0.74	0.31	0.26	0.07	0.24	0.12	0.32	0.15
37	0.46	0.09	0.03	0.08	0.34	0.12	0.37	0.20	0.13	0.19	0.11	0.12
38	0.92	0.03	0.01	0.01	0.04	0.00	0.81	0.06	0.05	0.05	0.02	0.02
39	0.19	0.04	0.08	0.22	0.47	0.07	0.71	0.06	0.07	0.10	0.06	0.20
40	0.73	0.04	0.02	0.00	0.20	0.39	0.83	0.06	0.06	0.01	0.04	0.42
41	0.09	0.05	0.07	0.10	0.70	0.23	0.07	0.16	0.21	0.25	0.32	0.11
42	0.36	0.03	0.11	0.05	0.44	0.09	0.37	0.16	0.18	0.18	0.10	0.02
43	0.09	0.04	0.04	0.01	0.82	0.22	0.11	0.17	0.22	0.06	0.44	0.04
44	0.48	0.14	0.05	0.01	0.32	0.31	0.83	0.07	0.04	0.02	0.04	0.47
45	0.97	0.00	0.01	0.00	0.02	0.00	0.87	0.04	0.05	0.02	0.02	0.01
46	0.27	0.07	0.00	0.19	0.47	0.31	0.78	0.09	0.00	0.09	0.05	0.40
47	0.74	0.01	0.10	0.05	0.11	0.05	0.81	0.04	0.06	0.06	0.02	0.02
48	0.17	0.01	0.06	0.00	0.75	0.54	0.28	0.08	0.21	0.00	0.43	0.36
49	0.18	0.16	0.04	0.07	0.55	0.43	0.72	0.10	0.05	0.07	0.06	0.52
50	0.19	0.01	0.08	0.00	0.72	0.21	0.26	0.07	0.25	0.05	0.36	0.10
**Mean (Pollard et al. [28])**						**0.23**	**Mean (Link et al. [29])**					**0.22**
